# Recent Advances in Nanoparticle-Mediated Delivery of Anti-Inflammatory Phytocompounds

**DOI:** 10.3390/ijms18040709

**Published:** 2017-03-28

**Authors:** Raffaele Conte, Valentina Marturano, Gianfranco Peluso, Anna Calarco, Pierfrancesco Cerruti

**Affiliations:** 1 Institute of Agro-Environmental and Forest Biology (IBAF-CNR), Via Pietro Castellino 111, 80131 Napoli, Italy; raffaele.conte86@tiscali.it (R.C.); gianfranco.peluso@ibaf.cnr.it (G.P.); 2 Institute for Polymers, Composites, and Biomaterials (IPCB-CNR), Via Campi Flegrei 34, 80078 Pozzuoli (NA), Italy; valentina.marturano@unina.it (V.M.); cerruti@ipcb.cnr.it (P.C.); 3 Department of Chemical Sciences, University of Naples “Federico II”, Via Cynthia 4, 80125 Napoli, Italy

**Keywords:** inflammation, phytochemicals, nanosized delivery systems, polyphenols, carbohydrates, cannabinoids, terpenoids, essential oils, nanoparticles, nanocapsules

## Abstract

Phytocompounds have been used in medicine for decades owing to their potential in anti-inflammatory applications. However, major difficulties in achieving sustained delivery of phyto-based drugs are related to their low solubility and cell penetration, and high instability. To overcome these disadvantages, nanosized delivery technologies are currently in use for sustained and enhanced delivery of phyto-derived bioactive compounds in the pharmaceutical sector. This review focuses on the recent advances in nanocarrier-mediated drug delivery of bioactive molecules of plant origin in the field of anti-inflammatory research. In particular, special attention is paid to the relationship between structure and properties of the nanocarrier and phytodrug release behavior.

## 1. Introduction

**Figure 1:**
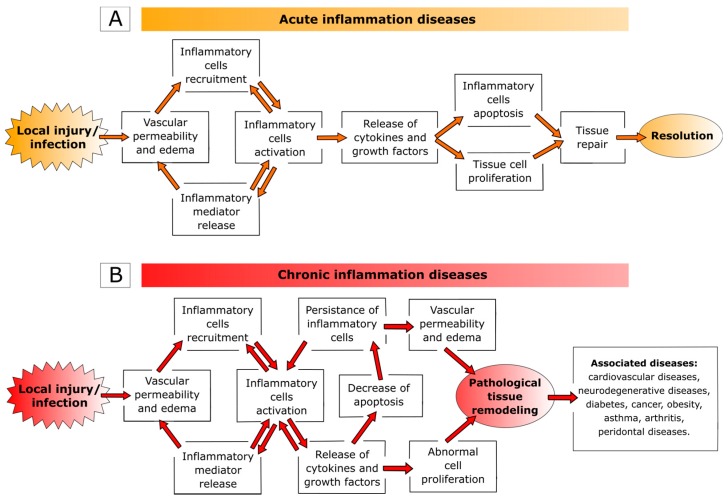
Schematic representation of the main characteristics of: acute (**A**) and chronic (**B**) inflammation.

Inflammation represents the physiological response of the body to tissue injury (e.g., stress, irritants, and radiations), infections (microbial and viral) or genetic changes and may be distinguished into two phases: acute and chronic. The acute is the early nonspecific phase characterized by local vasodilatation, increased capillary permeability, accumulation of fluid and blood proteins into the interstitial spaces, migration of neutrophils out of the capillaries, and release of inflammatory mediators (e.g., cytokines, lymphokines, and histamine) [1,2,3,4,5,6]. Clinically, acute inflammation is characterized by five cardinal signs: *rubor* (redness), *calor* (increased heat), tumor (swelling), *dolor* (pain), and *functio laesa* (loss of function). Under normal conditions, neutrophils undergo apoptosis after performing their action at the inflamed site and macrophages ingest apoptotic neutrophils. Clearance of apoptotic neutrophils prompts a switch from a pro- to an anti-inflammatory macrophage phenotype, which enable macrophage egress favoring tissue repair and regain of physiological function (Figure 1A) [7,8,9,10,11,12].

However, if the condition causing the damage is not resolved, the inflammatory process evolves toward subacute/chronic inflammation characterized by immunopathological changes such as infiltration of inflammatory cells, overexpression of pro-inflammatory genes, dysregulation of cellular signaling and loss of barrier function. The chronic state of inflammation has important roles in the onset of various diseases, including cardiovascular disease, neurodegenerative diseases, diabetes, cancer, obesity, asthma, as well as classic inflammatory diseases (e.g., arthritis and periodontal diseases) (Figure 1B) [13,14,15,16,17,18,19,20,21,22,23]. In the last decade, a new family of clinical disorders, namely systemic auto-inflammatory diseases, emerged. Auto-inflammatory disorders are a group of heterogeneous disorders characterized by apparently inexplicable recurrence of local inflammation, with an increased production of inflammatory cytokines that can occur without detectable autoantibodies or auto-reactive T cells. All these diseases are caused by overproduction of pro-inflammatory cytokines, such as interleukin (IL)-1β and tumor necrosis factor (TNF)-α, abnormal microbial sensing (as in inflammatory bowel disease), and tissue micro-damage leading to a pathological delay in the turning off of inflammatory responses [24,25,26].

Several evidences suggest that the regulation of inflammation is tightly controlled by an array of epigenetic mechanisms and cannot be confined to a simple model in which the expression of a distinct set of genes depends exclusively on a single or a defined set of transcription factors [27,28].

Suppression or inhibition of inflammatory/pro-inflammatory mediators using synthetic anti-inflammatory compounds (e.g., steroidal and nonsteroidal) is one of the major routes for the treatment of inflammatory disorders. However, the use of synthetic anti-inflammatory molecules is associated with some common side effects including gastric irritation, ulceration, bleeding, renal failure, hepatic failure, headache, hemolytic anemia, asthma exacerbation, skin rashes, angioedema, and pruritus [29,30]. In recent years, phytochemicals derived from plants have gained increased attention due to their safe toxicological profiles compared to allopathic drugs in the treatment of inflammatory disease [31,32,33,34], and their protective action has been demonstrated in obesity-mediated inflammation [35,36], inflammatory bowel disease [37,38,39,40], Alzheimer disease [41,42], diabetes [43], skin inflammation [44,45]. However, despite promising biological activities of plant raw extracts, poor solubility, poor stability, short biological half-life, and rapid elimination hinder their clinical application. Moreover, their large molecular size negatively affects passive absorption, and their pharmacokinetics is further modified by the highly acidic gastric pH and pre-systemic metabolism [46]. These aspects, together with the individual variation of extracts constituents, led research interest towards the synthesis of systems able to effectively deliver these substances.

## 2. Nanosized Drug Delivery Systems

Nanotechnology is already employed in traditional drug delivery and tissue engineering [47], as it offers the possibility to develop devices particularly adapted to improve the therapeutic efficacy of natural bioactive molecules [48]. Indeed, nanocarriers are alternatives for traditional formulation approaches, enabling to increase drug bioavailability, permit a site-specific targeted delivery, as well as reducing toxic side effects [49,50,51].

Since the 1970s, when Nobel Prize winner Christian de Duve described the structure and properties of lysosomes in biological tissues, drug administration protocols have significantly evolved due to the introduction of nanosized drug delivery systems [52]. The latter can be defined as ultra-dispersed solid organic or inorganic structures displaying a sub-micrometer size, typically comprised between 10 and 100 nm. The upper limit is dictated by the vector’s ability to pass cellular interstices, while the lower limit is fixed by the threshold for first-pass elimination by kidneys. Moreover, such dimensions permit a good biodistribution of long-circulating nanocarriers [53]. If compared to traditional formulations, the use of nanovectors for drug delivery offers many advantages: more efficient delivery of water insoluble drugs at high dose, protection for the drug from hostile environments (e.g., acidic pH in the digestive tract), and targeted and controlled delivery to achieve precise administration to a specific tissue over a determined period of time [54].

**Figure 2:**
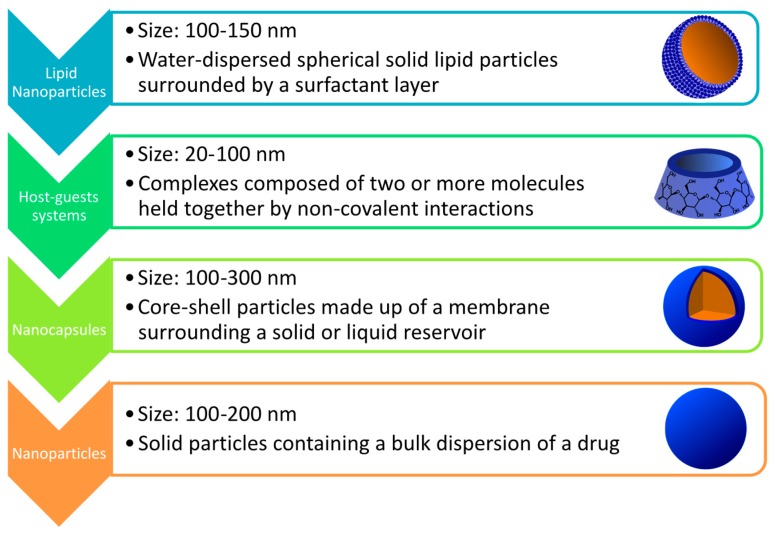
Examples of organic material-based nanosized drug delivery systems.

Various materials and structures have been employed as nanocarriers for either passive or active targeting. Metal particles, such as iron oxide or gold nanosized assemblies, can be surface modified to act as drug carriers [55,56]. However, organic materials are more versatile, and their properties and performances can be finely tuned by tailoring their chemical composition, size, shape, structures morphology, and surface properties [57], as depicted in Figure 2. Lipid-based particles, such as liposomes, or cubosomes, are supramolecular assemblies of amphiphilic molecules, in which hydrophobic drugs can be encapsulated [58,59]. Another class of natural carriers employed in drug delivery is cyclodextrins (CD), cyclic oligosaccharides characterized by a hydrophilic outer surface, and able to load guest molecules in their lipophilic inner cavity via non-covalent inclusion interactions. As well as other nanocarriers, the use of CD can increase drug solubility, bioavailability, safety, and stability of drug formulations [60]. Polymers provide even more versatility in nanocarriers engineering, being nanoparticles (NP), nanocapsules (NC), dendrimers and copolymer micelles among the numerous examples of polymer-based assemblies that serve as a reservoir for the loaded drug [61]. In nanoparticles and nanocapsules, drugs can be encapsulated in or conjugated to polymer chains. Dendrimers are made up of several branched polymer chains stemming from a central core, in which the active principle can be conjugated or complexed. Polymer micelles result from the self-assembly in water of amphiphilic copolymers into a core–shell structure. The hydrophobic core can be act as a reservoir of hydrophobic drugs while the hydrophilic corona provides water solubility and colloidal stability [62].

Tailored engineering and design of polymeric carriers allow precise and controlled administration of the cargo drug. A passive delivery occurs when the loaded drug is released by diffusion or erosion of the nanovector, an active delivery can instead be achieved employing stimuli-responsive materials as drug carriers. In stimuli-responsive (or smart) materials, a variation in structure or morphology is triggered by an external stimulus, such as pH variations, contact with concentrated ionic solution or enzymes, exposition to light, ultrasound, electric field, magnetic field or heating [63].

In this frame, the surface properties of the nanocarrier determine the interaction with body environment to a great extent [64]. For example, nanovectors with negative or positive surface charges have increased reticuloendothelial clearance. Steric stabilization with biological or synthetic macromolecules, such as opsonin or polyethylene glycol (PEG), prolongs circulation times and prevents nanoparticle loss [65]. Targeting strategies are aimed to overcome side effects and minimize systemic drug administration. These are classified into passive and active targeting [66]. Passive targeting is achieved by changing the physiochemical characteristics, pH or hydrophobicity of NP and utilizes the “enhanced permeability and retention effect” of inflamed blood vessels. Active targeting, differently, uses biomarkers (e.g., mutant genes, RNAs, proteins, lipids, carbohydrates, and small metabolite molecules) to reach the target sites [67]. The addition of a targeting moiety onto the surface of NP increases selective cellular binding and internalization through receptor-mediated endocytosis [53]. In this regard, key properties of nanoparticles (NP) utilized as plant-derived anti-inflammatory delivery systems greatly depend on size, surface characteristics, and targeting strategies.

Even though the use of nanocarriers as drug-delivery systems offers many advantages, some drawbacks need to be addressed, e.g., instability during blood circulation, low renal clearance, limited accumulation in specific tissues, and low uptake by target cells. Moreover, case by case evaluation of the interactions between nanocarriers and biological systems is of key importance to assess the reliability of the delivery systems. The successful translation of nano-formulations to the clinic involves careful assessment of their safety profiles, which, among other end-points, includes the evaluation of immunotoxicity [68]. Moreover, since physico-chemical properties of nanoparticles, such as surface charge and size, modulate uptake and interactions with cells, control over surface modification and biodegradation of nanovectors contributes to minimize potential health risks that are associated with occupational exposure to such materials [69]. The main challenges associated with nano-toxicity evaluations are related to the development of more accurate and fully-characterized reference materials and methods. In addition, there is the need to develop a paradigm for predictive toxicological testing of nanomaterials, taking into consideration the physico-chemical properties of the material that lead to molecular or cellular injury, with the aim to triage novel nanomaterials for further in vivo testing [70].

**Figure 3:**
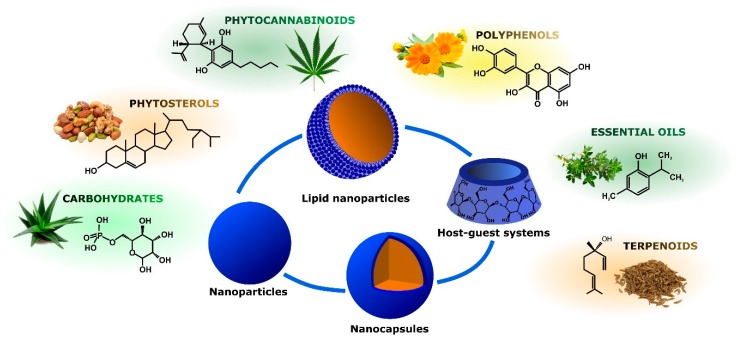
Nanoparticle-mediated delivery of different classes of anti-inflammatory natural compounds.

This review provides a focus on recent advances in the preparation and characterization of nanosized vectors able to release phytochemicals endowed with anti-inflammatory activity. Different delivery technologies will be discussed, according to chemical structure and natural source of the bioactive molecules to be released. In particular, organic nanovectors loaded with polyphenols, phytocannabinoids, phytosterols, carbohydrates, essential oils, and terpenoids will be reviewed (Figure 3). For each phytochemical class, a focus will be given to the relationship between structure and properties of nanocarriers and phytodrugs release behavior.

## 3. Anti-Inflammatory Phytoconstituents and Their Nanoparticle-Mediated Delivery

### 3.1. Polyphenols

**Figure 4:**
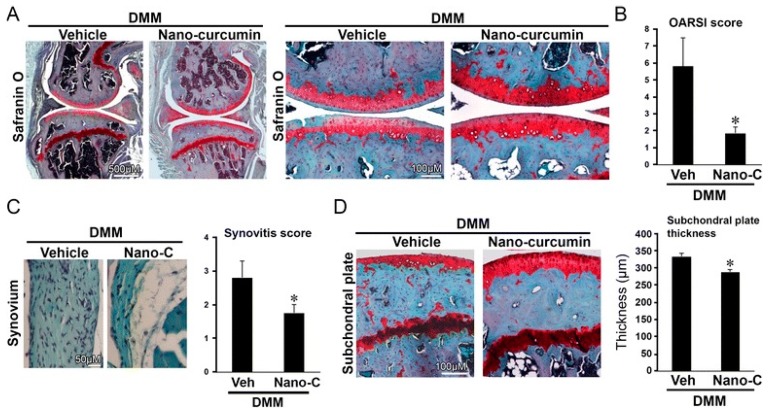
Topical application of nanoencapsulated curcumin slowed the progression of osteoarthritis induced by destabilization of the medial meniscus (DMM) in mice. Mice with DMM were treated daily with topical application of curcumin nanoparticles or vehicle. Mice treated topically with curcumin nanoparticles (Nano-C) exhibited improved Safranin O staining (**A**); lower Osteoarthritis Research Society International (OARSI) scores (**B**); and reduced synovitis (**C**); and subchondral bone plate thickness (**D**) at eight weeks after surgery compared to that in vehicle control (Veh) (* *p* < 0.05, *t*-test, *n* = 5/group). Reproduced from [96] (https://www.ncbi.nlm.nih.gov/pmc/articles/PMC4891896).

Polyphenols (PPH) are a large family of ubiquitous and varied molecules present as secondary metabolites in numerous vascular plants. These natural compounds range from simple molecules to complex structures that have in common the presence of benzene rings bearing one or several hydroxyl functions. In the plant, these active principles play an important role in growth, reproduction, resistance to pathogens, predators and diseases [71]. Nonetheless, PPH are potent effectors of biologic processes associated with the pathogenesis of human diseases thanks to their ability to interact with proteins, enzymes and membrane receptors, modulating the resulting activity [72]. Among their properties, the strong free radical scavenging action is probably the most studied, and is involved in the anti-inflammatory action of these molecules. In particular, quercetin (QC), resveratrol (RE) and tannins are recognized as pain killers [73]. PPH are able to improve health by strengthening the response of the immune system towards chronic diseases. However, their efficacy depends on their metabolism and bioavailability, defined as the amount of the administrated drug that is able to reach the systemic circulation. In general, PPH have a relatively low bioavailability due to both intrinsic factors (chemical structure, molecular weight and low hydrosolubility) and extrinsic factors (low stability in the gastrointestinal environment, extensive phase II metabolism and rapid elimination) [74]. Consequently, clinical applications of PPH are limited. To avoid these drawbacks, nanodelivery systems able to maintain the structural integrity of these bioactive molecules have been developed [75]. For example, nanosized vectors encapsulating QC, a semi-lipophilic flavonol ubiquitous in plants, have been developed by Li et al. through the synthesis of solid lipid nanoparticles (SLN) of 155 nm of size composed of soya lecithin, Tween-80 and polyethylene glycol (PEG), with a QC encapsulation efficiency of 91% [76]. These NP were able to increase the relative oral bioavailability of quercetin by 5.7-fold as compared to the free form. Lipid-coated nanocapsules (NC) have been reported by Barras et al., with a solubility 100 times higher with respect to free quercetin, stable for more than ten weeks, and with no degradation product being detected [77]. Pool et al. produced quercetin-loaded polylactic-co-glycolide (PLGA) NC with a more potent antioxidant action against peroxyl radical-induced lipid peroxidation, resulting in a more effective anti-inflammatory therapy [78]. Similarly, Wu et al. synthesized quercetin-loaded Eudragit-polyvinyl alcohol NP with particle size of 85 nm, good polydispersity, drug loading of around 99% and enhanced antioxidant activity [79]. Finally, Chakraborty et al. examined the potency of orally administered quercetin-PLGA NP in a rat model [80]. Nanoparticles were administered prior to alcohol induced gastric ulcer in the protection against oxidative damages, demonstrating that QC-PLGA NP prevented 90% of this inflammation as compared to the 20% of the free quercetin. Resveratrol, chemically known as 3,5,4-trihydroxystilbene, is a naturally occurring polyphenol produced by a wide variety of plants in response to injury, UV irradiation, ozone exposure and fungal attack [81]. Nanovectors delivering RE have been reported by Singh and Pai, that described a sustained release of trans-resveratrol from orally administered PLGA NP (drug encapsulation efficiency more than 78%, with a particle size of about 170 nm) [82]. The same authors encapsulated RE in Eudragit RL 100 NP with a drug incorporation efficiency of 84% and average size of 180 nm. In vivo studies in a rat model showed prolonged plasma levels up to 16 h, in comparison with free drug being cleared within 6 h [83]. Zu et al. developed carboxymethyl chitosan NP as carrier for resveratrol [84]. These nanoparticles (155 nm-sized, with an encapsulation efficiency of 44%) improved the solubility of resveratrol, thereby greatly affecting the antioxidant activity of the drug. Additionally, resveratrol loaded SLN were synthesized with a controlled release profile, due to an initial burst release of 40% caused by the active principle associated with the particle shell, and a subsequent prolonged release of the drug located in the lipid matrix. In this system, the efficiency of the cellular uptake depended on the molecular interactions with the biological membrane organization, lipid rafts and the actin cytoskeleton invaginations for the receptor mediated entrance [85]. Resveratrol loaded SLN have been also prepared by Pandita et al. with a drug incorporation efficiency of 89% and average diameter of 134 nm [86]. This drug delivery system showed prolonged release in vitro up to 120 h in a Wistar rat model, enhancing plasma bioavailability compared to free drug suspension. Finally, cyclodextrins-resveratrol complexes have been used to increase concentration of polyphenol in aqueous solution, while maintaining its biological activity. For example, spherical cyclodextrin-based nanosponges showed increasing solubility and stability, together with good drug encapsulation efficiency, compared to free RE [87]. Ellagitannin (ET) and ellagic acid (EAC) are active substances belonging to the phenolic class of tannins most present in pomegranate (*Punica granatum*), a fruit-bearing shrub that originated in the region from Iran to northern India [71]. Nanosized drug delivery systems protect these molecules inside the nanoparticulate core, thus preventing degradation and increasing bioavailability. For example, Bala et al. synthesized ellagic acid-loaded PLGA NP which showed a rapid initial release of EAC in pH 7.4 phosphate buffer, followed by a slower sustained release [88]. Further, the authors tested the influence of the stabilizers p-dimethylaminobenzaldehyde (DMAB) and polyvinyl alcohol (PVA) on size, loading efficacy, release kinetics in PBS, stability, cytotoxic activity and in situ intestinal permeability of these nanoparticles [88]. A similar study was conducted also on ellagic acid-loaded PLGA-polycaprolactone (PCL)NP [89]. Both investigations resulted in improved tannin bioavailability with potential therapeutic applications. In the last few decades, there has been considerable interest in the active compounds in turmeric called curcuminoids, among which the most abundant is curcumin (90%), studied for its antioxidant, anti-inflammatory, anticancer, antiviral, and antifungal properties [90]. However, a very small percentage of the orally administered curcumin is detected in blood plasma, while the vast majority is eliminated in the feces and the urine [67]. Several recent in vitro studies have suggested that the anti-inflammatory action of curcumin may be linked to its ability to limit the production of inflammatory mediators [91,92,93], inhibit extracellular matrix degradation and chondrocyte apoptosis [94], and act as an anti-oxidant by decreasing the over-production of reactive oxygen species [95]. Zhang et al. demonstrated in vivo that topical application of curcumin NP was efficacious not only in slowing osteoarthritis disease progression, but also on pain relief [96]. Using a post-traumatic osteoarthritis mouse model, they showed that the topical application of curcumin encapsulated in hydrogel/glass based NP preserves the chondroprotective activity of curcumin, and may increase its bioavailability (Figure 4).

Wang et al. reported an increment in curcumin anti-inflammatory activity when formulated in oil in water (o/w) nanoemulsions [97]. Emulsion-based formulations have been vastly employed in pharmaceutical, cosmetic and food industry to protect active ingredients against external factors, to enhance their stability, and to mask bad odors or taste [98]. Liposomes have also been employed as nanovectors for curcumin encapsulation and release [99]. Encapsulation of curcumin in liposomes, obtained using commercially available lecithin, was reported by Takahashi et al. [100]. Results showed that encapsulated curcumin was characterized by increased bioavailability, faster pharmacokinetics and better absorption of the drug in rats. Overall, Table 1 summarizes the nanosized delivery systems used for polyphenols with anti-inflammatory properties.

### 3.2. Phytocannabinoids

Phytocannabinoids are defined as the lipophilic substances deriving from the hemp plant, *Cannabis sativa* L., (Cannabaceae). The cannabis extract contains more than 460 compounds of which around 70 are phytocannabinoids. Among these, the primary psychoactive phytocannabinoid is Δ-9-tetrahydrocannabinol, commonly known as Δ9-THC [101], while other analogs available are cannabidiol (CBD), cannabigerol, Δ-9-tetrahydrocannabivarin, cannabidavirin and cannabinol. In particular, Δ9-THC and CBD are the main components of Sativex^®^, a marketed medicine for the treatment of cancer pain [102]. Phytocannabinoids act by modulating cannabinoid receptors, which are involved in fundamental physiological processes of central and autonomic nervous systems, immune, endocrine, reproductive and cardiovascular activity [103]. Moreover, phytocannabinoids link other receptors like the potential vanilloid type-1 receptor, the deorphanized G protein-coupled receptor, the peroxisome proliferator-activated receptors and the T-type Ca^2+^ channel. All these targets regulate the anti-nociceptive and anti-inflammatory response (Figure 3) but are also involved in the control of sleep [104], obesity [105], epilepsy [106], breast cancer cell migration [107], and lipid and glucose metabolism [108]. Further, innumerable therapeutic indications have been reported for phytocannabinoids such as analgesic, anticonvulsant, hypnotic, tranquilizer, anesthetic, anti-inflammatory, antibiotic, antiparasite, antispasmodic, nausea relieving, appetite stimulant, diuretic, antitussive and expectorant [109]. However, cannabis extract is today considered as a potential drug of abuse, and medicine uses nanotechnology to specifically target the active principle avoiding the psychotropic action and other side effects. For example, Esposito et al. described the development and optimization of a method to encapsulate phytocannabinoids in nanostructured lipid carriers [110]. Such vectors were produced by a melt and ultrasonication protocol specifically designed to optimize nanoparticle recovery and drug encapsulation efficiency [110]. Similarly, Duran Lobato et al. proposed a valuable oral delivery carrier for cannabinoid derivatives against chronic pain states based on lipid NP formulations [111]. Such vectors were developed through solvent-emulsion evaporation, and optimized regarding the physicochemical properties, long-term stability, and integrity under simulated gastric conditions. In particular, the selection of the lipid core and the inclusion of lecithin proved to be key factors for the final properties of encapsulation, integrity, and performance of the carriers [111]. The same authors prepared surface modified PLGANP. In particular, plain and surface-modified particles were successfully developed using a nanoprecipitation method, using chitosan (CS), Eudragit RS, lecithin and vitamin E as surface modifying agents. The nanoparticles exhibited mean particle size distributions in the range of 253–344 nm, spherical shape and controlled zeta potential values. High values of entrapment efficiency were obtained for all the formulations, while lecithin and vitamin E-modified particles showed higher release rates when compared to the other formulations [112]. Hernán Pérez de la Ossa et al. developed cannabidiol loaded PCL particles as a suitable dosage form, prepared by the oil-in-water emulsion–solvent evaporation technique [113]. These vectors had high entrapment efficiency (around 100%). CBD dissolved in the polymeric matrix of the microspheres was slowly released in vitro within ten days. The results suggested that PCL particles were an alternative delivery system for long-term cannabinoid administration [94]. Martín-Banderas et al. produced Δ9-THC-loaded PLGA nanoparticles [114]. The nanoformulation was further improved by surface functionalization with CS and PEG, to optimize the pharmacokinetics. Encapsulation efficiency (around 95%) was not affected by the type of polymeric coating. However, CS and PEG coatings decreased and increased, respectively, the Δ9-THC release kinetics. The safety of all PLGA-based formulations was confirmed by in vivo compatibility studies; moreover, protein adsorption tests suggested that PEGylation protected the phytodrug against immune processes [114]. Table 2 acts as a summary of synthesized nanosized delivery systems for phytocannabinoids.

### 3.3. Phytosterols

Phytosterols (plant sterols and stanols) are naturally occurring compounds ubiquitously found in vegetable oils, cereal grains, nuts, legumes, fruits, and vegetables. In particular, the term phytosterol refers to more than 200 different compounds among which sitosterol, campesterol, sitostanol and campestanol are the most diffused. Phytosterols have various bioactive properties such as blood cholesterol-lowering effect via partial inhibition of intestinal cholesterol absorption, anti-atherogenic effects and, particularly for β-sitosterol, immune stimulating and anti-inflammatory activities. Furthermore, several studies have suggested the beneficial effects of plant sterols against the development of different types of cancer, such as colorectal, breast and prostate cancer [115]. Despite their structural similarity to cholesterol, their bioavailability is limited. In particular, absorption is less than 2% for phytosterols, while it is 30–60% for cholesterol. On this basis, the use of nanotechnology can dramatically contribute improving the phytosterols pharmacokinetics. To this aim, Leong et al. prepared and characterized water-soluble phytosterols nanodispersions. In particular, he studied the effects of four different types of organic phases (hexane, isopropyl alcohol, ethanol, and acetone), their ratio with the aqueous phase and the use of conventional homogenization vs. high-pressure homogenization on the vectors obtained. Phytosterol nanodispersions were finally produced by emulsification-evaporation using hexane [116]. Rossi et al. synthesized phytosterols colloidal particles using antisolvent precipitation in presence of a non-ionic surfactant. The resulting colloidal particles had rod-like shape with some degree of crystallinity. Moreover, the colloidal dispersions had good stability ensured by surface charge, due to the presence of water and hydroxyl groups on the particle surface, and by steric stabilization, due to the use of a non-ionic stabilizer [117]. Turk et al. produced stable suspensions of submicron particles of phytosterol by rapid expansion of a supercritical solution into aqueous solution using four different surfactants to impede growth and agglomeration of the submicron particles. The obtained sizes were about 500 nm and, in most cases, a bimodal particle size distribution was obtained. Long-term stability studies indicated modest particle growth over 12 months [118]. Table 3 shows examples of nanosized delivery systems for anti-inflammatory phytosterols.

### 3.4. Carbohydrates

Carbohydrates are one of the major components of plants and are essential to the proper growth and development of vegetables. In fact, carbohydrate components make up 90% of the primary cell wall and are critical to wall function. Three types of polysaccharides, i.e., high molecular weight carbohydrates, mainly contribute to plant structure: pectin, hemicelluloses and cellulose. Pectins are most abundant in the plant primary cell walls and in the middle lamellae, and are defined by the presence of galacturonic acid. Hemicelluloses and cellulose represent the pure structural portion of the plants and are differentiated by the fact that hemicelluloses are small, branched carbohydrate compounds made of different monosaccharides, while cellulose is made of long unbranched fibrils composed exclusively of glucose, held together by hydrogen bonds. Moreover, hemicellulose is synthesized in the Golgi, while cellulose is synthesized in the cytoplasm, near the plasma membrane [119]. Many plant polysaccharides have a strong anti-inflammatory action. For example, such as *Echinacea purpurea* and *Echinacea angustifolia* (Asteraceae) that are known for their immunostimulating and skin repairing properties [120]. Aqueous fractions obtained from the roots of these asteraceae contain echinacin, a polysaccharide with promising anti-inflammatory activity in the skin of mice subjected to croton oil induced inflammation. A pectin isolated from the aerial parts of *Comarum palustre* (Rosaceae), the comaruman, was found to possess immunomodulating and anti-inflammatory activity. In particular, the galacturonan fragments obtained by partial hydrolysis of pectin (with molecular weight up to 10 kDa) were able to decrease in vitro the adhesion of neutrophils to fibronectin to a greater extent than the parent pectin [121,122]. Popov et al. demonstrated a preventive effect of comaruman on acetic acid-induced colitis in mice [123]. The oral administration of this pectin for two days prior to induction of colitis reduces neutrophil infiltration and enhances colon-bound mucus compared with the vehicle-treated colitis group.

The sulfated polysaccharides like xylose, glucose, arabinose, galactose and galactosamine present in *Artemisia tripartita* (Asteraceae) showed anti-inflammatory properties due to their ability to alter macrophage function, neutrophil count, and complement fixation function [124]. However, the most recent studies on nano-delivery of anti-inflammatory carbohydrates deal with the fresh extract of *Aloe vera*, a succulent plant native to northern Africa that has rapidly spread across the world [125]. In particular, the fresh gel mainly consists of water (99.1%) and mesophyll cells (0.9% dry matter) with the predominant sugar represented by mannose as mannose-6-phosphate, followed by other sugars in varying concentrations. Overall, arabinose, xylose, mannose, galactose, and glucose account for 69.2% of the total [125]. Liposomes containing *Aloe vera* gel having intrinsic anti-inflammatory action, and with particle size of 200 nm have been prepared by Takahashi et al. to enhance skin collagen synthesis and growth of skin cell lines [126]. Encapsulation and stabilization of *Aloe vera* extract on cotton fabric for wound dressing application has been recently accounted for [127]. Moreover, co-encapsulation of *Aloe vera* with curcumin enhanced the delivery of the phytocompounds and their antioxidant activity [128]. Esmaeili et al. prepared polyamide NC containing *Aloe vera* by the emulsion diffusion technique. In particular, sebacoyl chloride, *Aloe vera* extract and olive oil were dissolved in a complex organic phase made of acetone, ethyl acetate and dimethyl sulfoxide, while diethylenetriamine was solubilized in water to form the aqueous phase. Tween and gelatin were used as stabilizers. Nanocapsule dimensions depended on the polymer to oil ratio, the relative amount of polymers and plant extract and the use of surfactants. Optimized conditions resulted in nanocapsules of 115 nm [129]. Table 4 summarizes the available nanosized vectors for carbohydrates with anti-inflammatory activity derived from plants.

### 3.5. Essential Oils

Essential oils (EOs) are hydrophobic, volatile, natural, complex compounds characterized by a strong odor, formed by aromatic plants as secondary metabolites. Traditional EOs extraction techniques, usually based on steam or hydro-distillation, date back to the middle ages, when they were developed in Arabic Spain. Known for their antibacterial [130] and fungicidal [131] activity, and for their fragrance and flavor, they are also employed in food preservation [132] and in medicine as analgesic, sedative, anti-inflammatory, spasmolytic and local anesthetic remedies [133]. EOs have been extensively studied, and there is much literature regarding their desirable properties, however they are also very sensitive to the effects of light, oxygen, humidity and high temperature, so that their applications are limited [134]. Novel technologies for the administration of plant extracts may have remarkable advantages over conventional formulations in terms of enhancement of solubility, bioavailability, protection from toxicity, enhancement of pharmacological activity, enhancement of stability, improved tissue macrophages distribution, sustained delivery, and protection from physical and chemical degradation [135]. Parris et al. have reported the encapsulation of oregano and cassia pure EOs in corn zein NC via phase separation method, resulting in a fast and high yield procedure [136]. Reportedly, EOs loaded particles have limited digestibility in the stomach, slow release in the small intestine, and more rapid release in the large intestine. It is worth noticing how, in their encapsulated form, little if no interaction of the EO with other components in the feed was found. Zein, a prolamine protein found in maize, known for its superior biodegradability and biocompatibility, has also been used to nanoencapsulate thymol and carvacrol EOs via liquid–liquid dispersion method [137]. One of the most promising methods for the solubilization and stabilization of natural active agents involves the use of cyclodextrins (CD) as carriers [138]. In aqueous solution, the slightly polar CD cavity is occupied by water molecules and therefore they can be readily substituted by appropriate “guest molecules”, which are less polar than water [139]. Cevallos et al. have performed studies on the encapsulation and release of thymol and cinnamaldehyde from CD [140]. The importance of this work lays in the studies concerning the influence of water adsorption by CDs on the release of encapsulated compounds. Results showed the relevance of selecting appropriated storage conditions for hydrophobic flavors encapsulated in CD or for predicting the shelf life of functional products formulated with nanoencapsulated compounds. Olivera et al. reported the preparation of alginate/cashew gum NP via spray-drying, aiming at the development of a biopolymer blend for encapsulation of *Lippiasidoides* essential oil, primarily composed of thymol (50–70%) [141]. In the last decades, encapsulation techniques have frequently been supported by innovative controlled and triggered release. Stimuli-responsive materials have therefore been employed as shell material for capsules and vector for drugs and other active agents [142]. Bizzarro et al. recently reported on the preparation of cumin and basil oil-loaded polyamide capsules able to release their cargo oil under UV-light irradiation [143]. Photo-responsive as well as other stimuli-responsive polymeric capsules have been vastly reviewed in the literature [144]. Table 5 summarizes the nanosized delivery systems proposed for anti-inflammatory essential oils.

### 3.6. Terpenoids

Among a wide spectrum of natural products, terpenoids play a significant role in prevention and treatment of anti-inflammatory conditions. These compounds are produced as secondary metabolites of different organisms, such as plants, mosses, algae, lichens, as well as insects, microbes and marine organisms [145]. In recent years, the molecular basis of the anti-inflammatory effect of plant-derived terpenoids has been at the center of extensive research. Several studies demonstrated that terpenoid ingredients can suppress nuclear factor-κB (NF-κB) signaling, the major regulator in the pathogenesis of inflammatory diseases and cancer [146]. Terpenoids are a very diverse group of molecules, with extraordinary varying chemistry, structure and function. These compounds are classified according to the number of isoprene units and carbon atoms [147]. Notably, the list of terpenes includes d-limonene, squalene, geraniol, linalool, artemisinin, *p*-cymene, thymol and β-carotene. Targeted delivery of typically hydrophobic terpenoids has also been the subject of massive research, finding applications in a wide variety of fields. Among natural terpenoids, squalene is one of the most widespread, present in olives, shark liver oil, wheat germ and even human skin cells, and possesses versatile applications [148]. Lacatusu et al. reported the design and characterization of soft lipid nanocarriers based on bioactive pumpkin and amaranth oils, rich in squalene, for co-encapsulation and co-delivery of UV-A and UV-B filters (avobenzone andoctocrylene) [149]. The same group later reported the preparation of squalene-based nanocarriers for the encapsulation and drug delivery of hydrophilic and lipophilic actives, confirming the protective and stabilizing effect of squalene as protective for highly sensitive molecules [150]. On the other hand, squalene can also be employed as active agent. Adamczak et al. developed a method of preparation of polyelectrolyte multilayer capsules, containing squalene as hydrophobic phase, by membrane emulsification technique [151,152]. In that work, squalene served as both active agent and solvent cargo for hydrophobic drug. Alongside its anti-inflammatory activity, squalene has been reported having in vitro cytoprotective activity [153], and has been found effective for *alopecia areata* treatment [154]. Lycopene is a carotenoid showing good pharmacological properties including antioxidant and anti-inflammatory, commonly found in tomatoes, red carrots, watermelons, and other red fruits and vegetables [155,156]. As such, lycopene has a limited systemic absorption due to a very low aqueous solubility. To overcome this drawback, lycopene nanosized delivery systems have been recently engineered. Butnariu and Giuchici developed nanoemulsions based on aqueous propolis and lycopene, which were tested as protective agents against acute UV-A-induced inflammation on rat paw. A better therapeutic effect was noticed compared to a standard suspension, coupled with extended time interval of tested parameters [157]. More recently, stable lycopene-loaded SLN based on myristic acid or orange wax have been described, highlighting that chemical stability of lycopene entrapped in the SLN was significantly enhanced [158,159]. As reported in the previous sections, cyclodextrins (CD) constitute one of the most reliable nanocarriers for hydrophobic molecules, such as essential oils and terpenoids. For example, a method to load lycopene into maltodextrin and cyclodextrin has been recently proposed by Seo et al. [160]. The comparison of the anti-inflammatory effect of nanoencapsulated and free lycopene on macrophage cell lines demonstrated that nanoencapsulation of lycopene can further improve its anti-inflammatory effect during tissue-damaging inflammatory conditions.

The interaction between β-CD and *p*-cymene, one of the main anti-inflammatory constituents of thyme and cumin EOs, has also been extensively studied [161,162]. Encapsulation of *p*-cymene in CD has been successfully reported by Quintans et al. [163]. This study involved the oral administration of *p*-cymene/CD inclusion complexes to inhibit mice edema. Interestingly, *p*-cymene/β-CD inclusion provided faster and more powerful inhibition than the same amount of free *p*-cymene at early stage, indicating that CD nanocarriers can improve *p*-cymene anti-nociceptive and anti-inflammatory effects. Other authors reported the encapsulation of other terpenoids in CD. Menezes et al. discussed the inclusion complex of β-CD and (−)-linalool [164], a monoterpene alcohol compound prevalent in essential oils of various aromatic plant species [165]. Other studies, carried out by Quintans-Júnior, confirmed that (−)-linalool-CD complexes produced superior anti-nociceptive effect, with respect to that of (−)-linalool alone, in experimental pain protocols [166]. In analogy with this result, Guimarães et al. reported that the encapsulation of carvacrol, a terpenoid phenol present in the essential oil of oregano, with β-CD, improves the pharmacological response on cancer pain experimental protocols [167]. It is worth mentioning that the terpene-cyclodextrin inclusion systems have been vastly reviewed in literature [168]. Overall, Table 6 recaps the main nanosized delivery systems for anti-inflammatory terpenoids.

## 4. Conclusions

**Figure 5:**
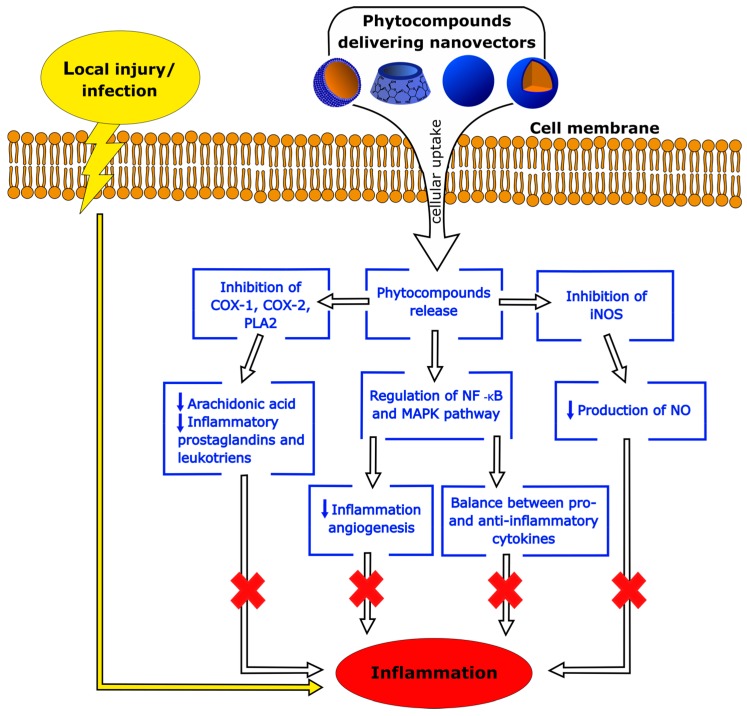
Schematic diagram of the phytocompounds effects on the main cellular pathways involved in inflammation. After cell internalization of a nanovector, encapsulated phytocompounds are released into cytoplasm. Their anti-inflammatory action is elicited via inhibition of nitric oxide (NO) production by nitric oxide synthase (iNOS); reduction of arachidonic acid metabolites and prostaglandins through inhibition of the cyclooxygenase (COX) and Phospholipase A2 (PLA2) pathways; and regulation of nuclear factor NF-κB and mitogen-activated protein kinases (MAPKs) pathways, which modulate the expression of pro- and anti-inflammatory mediators including cytokines, chemokines, and adhesion molecules.

Nowadays, phytochemicals have promising potential to prevent and/or treat inflammatory diseases. However, poor water solubility, stability and bioavailability, together with the side effects reported when used in therapy, have limited their clinical application so far. Nanosized drug delivery systems can increase the solubility and stability of phytochemicals, enhance their absorption, protect them from premature enzymatic degradation or metabolism in the body, and prolong their circulation time, limiting their side effects. A schematic diagram of the effects of nanoparticle-delivered phytocompounds on the main cellular pathways involved in inflammation is reported in Figure 5.

Nanotechnology represents a promising approach, which has already provided significant steps forward to bringing anti-inflammatory phytochemicals much closer to clinical applications. In this frame, the increase in the number of nanotoxicological studies in the literature is related to a growing awareness and sensibility towards the ethics of nanotechnology. Additional research is needed to improve the cost-effectiveness and long-term safety of nanosized drug delivery systems, through the use of biocompatible materials and un-harmful release processes, aimed to establish a stronger correlation between smart nanoparticle design and rigorous safety assessment for future applications in biomedicine.

## Figures and Tables

**Table 1:** Nanosized delivery systems for anti-inflammatory polyphenols.

Bioactive Principle	Nanovector	Type of Delivery System	Experimental Model	Reference
Quercetin 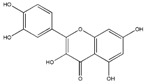	Nanoparticles	NP of soya lecithin, Tween-80 and PEG	In vivo (rats)	[76]
		PLGA NP containing quercetin	In vitro	[78]
		Eudragit-polyvinyl alcohol quercetin-loaded NP	In vitro	[79]
		Quercetin-PLGA NP	In vivo (rats)	[80]
	Nanocapsules	Lipid-coated NC	In vitro	[77,78]
		Quercetin-PLGA NC		
Resveratrol 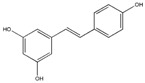	Nanoparticles	PLGA NP containing resveratrol	In vivo (Wistar male rats)	[82]
		Eudragit RL 100 NP	In vitro/In vivo	[83]
		Carboxymethyl chitosan NP	In vitro/In vivo (rats)	[84]
	Solid lipid nanoparticles	SLN with a controlled release profile	In vitro	[85]
		Resveratrol loaded SLN	In vivo (Wistar male rats)	[86]
	Cyclodextrins	CD-based nanosponges	In vitro	[87]
Ellagic acid 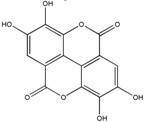	Nanoparticles	Ellagic acid-loaded PLGA NP	In vitro	[88]
		PLGA-PCL ellagic acid NP	In vivo (rats with induced nephrotoxicity)	[89]
Curcumin 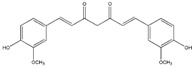	Nanoparticles	Hydrogel/glass	In vivo (rats with post-traumatic osteoarthritis)	[96]
	Nanoemulsions	o/w nanoemulsion containing curcumin in the oil phase	In vitro	[98]
	Lipid nanoparticles	Lecithin liposomes	In vivo (rats)	[100]

NP, nanoparticles; PEG, polyethylene glycol; PLGA, polylactic-co-glycolic acid; NC, nanocapsules; SLN, solid lipid nanoparticles; CD, cyclodextrins; PCL, polycaprolactone; o/w, oil in water.

**Table 2:** Nanosized delivery systems for anti-inflammatory phytocannabinoids.

Bioactive Principle	Nanovector	Type of Delivery System	Experimental Model	Reference
Δ-9-Tetrahydrocannabinol 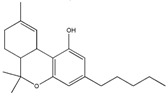	Lipid nanoparticles	Nanostructured lipid carriers	In vitro	[110]
	Nanoparticles	Lipid NP containing lecithin	In vitro	[111]
		PLGA NP	In vitro/In vivo	[112]
		Cannabidiol loaded PCL NP	In vitro	[113]
		Δ9-THC-loaded PLGA NP	In vivo (immunocompetent C57BL/6 mice)	[114]

**Table 3:** Nanosized delivery systems for anti-inflammatory phytosterols.

Bioactive Principle	Nanovector	Type of Delivery System	Experimental Model	Reference
Phytosterol 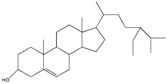	Nanodispersion	Nanodispersion produced by emulsification-evaporation	In vitro	[116]
		Colloidal particles using antisolvent precipitation	In vitro	[117]
		Suspensions of submicron particles of phytosterol	In vitro	[118]

**Table 4:** Nano sized delivery systems for anti-inflammatory carbohydrates.

Bioactive Principle	Nanovector	Type of Delivery System	Experimental Model	Reference
Mannose-6-phosphate 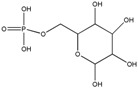	Liposomes	Liposomes containing *Aloe vera* gel	In vitro	[123]
		Liposomes containing *Aloe vera* gel co-encapsulated with curcumin	In vitro	[125]
	Nanocapsules	Polyamide NC	In vitro	[126]

**Table 5:** Nanosized delivery systems for anti-inflammatory essential oils.

Bioactive Principle		Nanovector	Type of Delivery System	Experimental Model	Reference
Oregano and Cassia EO		Nanoparticles	Corn zein NP	In vitro	[136]
Thymol and carvacrol		Nanoparticles	Corn zein NP	In vitro	[137]
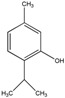	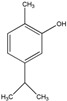				
Cinnamon and thyme EO		Cyclodextrins	Inclusion in CD	In vitro	[140]
*Lippiasidoides*EO (50–70% Thymol)		Nanoparticles	Alginate/cashew gum NP	In vitro	[141]
Cumin and basil EO		Nanocapsules	Polyamide NC	In vitro	[143]

**Table 6:** Nanosized delivery systems for anti-inflammatory terpenoids.

Bioactive Principle	Nanovector	Type of Delivery Systems	Experimental Model	Reference
Squalene 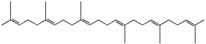	Nanocapsules	Polyelectrolyte multilayer NC	In vitro	[151]
Lycopene 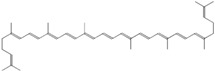	Nanoemulsions	Aqueous propolis and lycopene	In vivo (albino guinea pigs)	[157]
	Nanoparticles	SLN	In vitro	[158,159]
	Cyclodextrins	Inclusion in CD	In vitro	[160]
*p*-Cymene 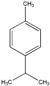	Cyclodextrins	Inclusion in CD	In vitro	[162,163]
(−)-Linalool 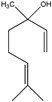	Cyclodextrins	Inclusion in CD	In vivo (rodents)	[164,166]
Carvacrol 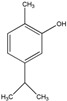	Cyclodextrins	Inclusion in CD	In vivo (rodents with induced tumors)	[167]

## Abbreviations

DDS	Drug delivery systems
PPH	Polyphenols
NP	Nanoparticles
NC	Nanocapsules
SLN	Solid lipid nanoparticles
QC	Quercetin
RE	Resveratrol
PLGA	Polylactic-co-glycolic acid
ET	Ellagitannin
EAC	Ellagic acid
PEG	Polyethylene glycol
PVA	Polyvinyl alcohol
DMAB	*p*-dimethylaminobenzaldehyde
PBS	Phosphate-buffered saline
PCL	Polycaprolactone
THC	Tetrahydrocannabinol
CBD	Cannabidiol
CS	Chitosan
EO	Essential oils
CD	Cyclodextrins
